# Changing lung function and associated health-related quality-of-life: A five-year cohort study of Malawian adults

**DOI:** 10.1016/j.eclinm.2021.101166

**Published:** 2021-10-18

**Authors:** Martin W. Njoroge, Patrick Mjojo, Catherine Chirwa, Sarah Rylance, Rebecca Nightingale, Stephen B. Gordon, Kevin Mortimer, Peter Burney, John Balmes, Jamie Rylance, Angela Obasi, Louis W. Niessen, Graham Devereux, IMPALA consortium

**Affiliations:** a Department of Clinical Sciences and International Public Health, Liverpool School of Tropical Medicine, Pembroke Place, Liverpool L3 5QA, UK; b Malawi Liverpool Wellcome Trust Programme, Blantyre, Malawi; c Liverpool University Hospital NHS Foundation Trust, Liverpool, UK; d National Heart and Lung Institute, Imperial College, London, UK; e University of California, San Francisco, United States of America; f University of California, Berkeley, United States of America; g John Hopkins Bloomberg School of Public Health, Baltimore, United States of America

## Abstract

**Background:**

In Sub-Saharan Africa cross-sectional studies report a high prevalence of abnormal lung function indicative of chronic respiratory disease. The natural history and health impact of this abnormal lung function in low-and middle-income countries is largely unknown.

**Methods:**

A cohort of 1481 adults representative of rural Chikwawa in Malawi were recruited in 2014 and followed-up in 2019. Respiratory symptoms and health-related quality of life (HRQoL) were quantified. Lung function was measured by spirometry.

**Findings:**

1232 (83%) adults participated; spirometry was available for 1082 (73%). Mean (SD) age 49.5 (17.0) years, 278(23%) had ever smoked, and 724 (59%) were women. Forced expiratory volume in one second (FEV_1_) declined by 53.4 ml/year (95% CI: 49.0, 57.8) and forced vital capacity (FVC) by 45.2 ml/year (95% CI: 39.2, 50.5) . Chronic airflow obstruction increased from 9.5% (7.6, 11.6%) in 2014 to 17.5% (15.3, 19.9%) in 2019. There was no change in diagnosed asthma or in spirometry consistent with asthma or restriction. Rate of FEV_1_ decline was not associated with diagnosed Chronic obstructive pulmonary disease (COPD), asthma, or spirometry consistent with asthma, COPD, or restriction. HRQoL was adversely associated with respiratory symptoms (dyspnoea, wheeze, cough), previous tuberculosis, declining FEV_1_ and spirometry consistent with asthma or restriction. These differences exceeded the minimally important difference.

**Interpretation:**

In this cohort, the increasing prevalence of COPD is associated with the high rate of FEV_1_ decline and lung function deficits present before recruitment. Respiratory symptoms and sub-optimal lung function are independently associated with reduced HRQoL.

**Research in context**

**Evidence before this study**

Multi-national studies report a high prevalence of abnormal lung function in low- and middle-income countries (LMICs) indicative of airflow obstruction (COPD, asthma) and restriction. Very few studies have conducted longitudinal measurement to investigate the natural history of lung function in LMICs and impact on health-related quality of life (HRQoL).

**Added value of this study**

This study in rural Malawi showed that in a general population of adults, the prevalence of COPD nearly doubled from 9.5% to 17.5% in 5 years. The rate of lung function decline in predominantly non-smoking adults is comparable with that reported for smokers of ≥15 cigarettes a day in high income countries. The respiratory symptoms and reductions in lung function experienced by adult Malawians are associated with clinically significant reductions in HRQoL.

**Implications of all the available evidence**

The high prevalence of COPD in sub-Saharan Africa adversely affects quality of life and is, in part, a consequence of accelerated lung function decline. The evidence justifies the implementation of sustainable initiatives for widespread diagnosis and management of chronic respiratory diseases in sub-Saharan Africa.

Alt-text: Unlabelled box

## 1 Introduction

Chronic obstructive pulmonary disease (COPD) and asthma are the most prevalent chronic respiratory diseases (CRDs) [1]. Globally, COPD accounts for three million deaths annually, 90% of which are in low- and middle-income countries (LMICs) [1,2]. Asthma is the most common CRD, affecting 360 million people, it is the commonest chronic disease of childhood and remains a burden to individuals, their families, and healthcare systems [1]. In LMICs, population-based surveys have reported a high prevalence of respiratory symptoms [3], [4], [5], obstructive and restrictive lung function [6,7], but low prevalence of diagnosed COPD [8] suggesting an unrecognised clinical need. International Guideline recommendations are that asthma and COPD require long term treatment and follow-up [9,10]. Knowing the true lifetime CRD burden may potentially have profound implications for provision of respiratory health services. However, given the scarcity of resources competing for healthcare priority there is a pressing need to better document the impact of CRD's on long-term general morbidity. Few studies have reported and described the general health impact of diagnosed (and undiagnosed) asthma and COPD in LMICs [11].

In Malawi, two studies have reported significant levels of abnormal lung function with restrictive and obstructive lung function deficits being present in 34.8% (95% CI: 31.7%, 38.0%) and 8.7% (7.0%, 10.7%) of rural adults, respectively, and 38.6% (34.4%, 42.8%) and 4.2% (2.0%, 6.4%) of urban adults, respectively [5,12]. Whether these lung function deficits arise from failure to attain the normal lung function plateau in the third decade and/or from subsequent accelerated decline of lung function remains to be ascertained. In addition, the impact of these lung function deficits on general health status, peoples’ everyday lives, and subsequent health service needs, is largely unknown in this and other LMICs settings.

We report here the findings of a cohort study in rural Malawi [3] in terms of annualised rate of change in lung function and associated impact on health-related quality of life (HRQoL) to investigate the natural history of abnormal lung function and its impact on HRQoL in a LMIC.

## 2 Materials and methods

### 2.1 Setting and study design

Malawi is one of the world's poorest countries (per capita GDP-$300) and 83% of its 18 million inhabitants live in rural areas [13]. The prevalence of diagnosed COPD and asthma in Malawi is unknown because lung function testing is unavailable out with research settings. Chikwawa (population 360,000) is a predominantly rural district of 4755 km^2^ in Southern Malawi.

We report the five year follow-up of a cohort of adult Malawians recruited in 2014. The Chikwawa lung health cohort has been described in detail elsewhere: Njoroge et al. [3]. In 2014 a community-based prevalence survey, nested within a cookstove intervention trial, was conducted in accordance with the Burden of Obstructive Lung Disease (BOLD) protocol [3,14]. Participants were randomly sampled from adults living in 50 villages in Chikwawa: [3,12] of the 3000 invited, 1481 participated. Non-participation was usually because of inability to make contact, non-participants were younger and more likely to be male [3,12].

Two rounds of follow-up in 2015 (*n* = 1090) and 2017 (*n* = 989) investigated whether lung function decline was affected by the cook stove intervention or personal air pollution exposures [15]. In 2019, all the participants from 2014 were invited to take part in this five year follow-up.

### 2.2 Ethical considerations

The study was approved by the Imperial College Research Ethics Committee (17IC4272), Liverpool School of Tropical Medicine Research Ethics Committee (19–005) and the Malawi College of Medicine Research and Ethics Committee (COMREC, P.03/19/2617). All participants provided written informed consent.

### 2.3 Data collection

Assessments in 2019 were almost identical to those in 2014, 2015 and 2017 and are described in detail elsewhere [3,12,15]. Interviewer administered questionnaires covered respiratory symptoms, smoking history, environmental exposures, demographics, socio-economic status, and diagnosed respiratory disease [3,12].

Lung function (forced expiratory volume in one second [FEV_1_] and forced vital capacity [FVC]) was measured by spirometry (NDD EasyOne Spirometer, NDD Medizintechnik AG, Switzerland), performed before and 15 min after 200 µg inhaled salbutamol via spacer. Standard contraindications for spirometry were applied and pregnant women were not tested. The trained and certified spirometry technicians received regular feedback on spirogram quality in accordance with the BOLD protocol [14] and ATS/ERS recommendations [16]. The quality of each spirometry trace was reviewed and scored based on acceptability and repeatability criteria [14,16]. FEV_1_ and FVC readings ranked A and B were analysed [16].

The 12–item Short Form Survey (SF–12) (administered in 2014) and the Veterans RAND 12 item survey (VR–12) (administered in 2019) were used to quantify HRQoL from responses to questions on general health perceptions, physical functioning, role limitations due to physical and emotional problems, bodily pain, energy and fatigue, social functioning, and mental health [17,18]. The validated Brazier SF–6D algorithm was applied to obtain a summary preference-based health utility measure (HRQoL utility score) that ranged from 0 (for death) to 1 (full health) [19]. VR–12 responses were mapped to the utility scores from the Brazier SF–6D algorithms to ensure that the scores derived from the SF–12 and VR–12 were comparable. We also assessed the distributional properties of scores obtained from SF–12 and VR–12 tools through the SF–6D algorithms to confirm that they were similar [20,21]. The 2015 and 2017 follow-ups did not include HRQoL.

### 2.4 Statistical considerations

In 2014, a randomized age, sex-stratified sample was identified from lists of adults living in the 50 cookstove trial villages [3,12]. The baseline sample size estimate of 1200 [3,12], based on the BOLD protocol [14], resulted in a minimum sample size of 300 in any one age/sex stratum (two age groups: 18–39years, ≥ 40years) required to approximate an expected prevalence of fixed airflow obstruction of 10–25% with a precision 3.3–5.0% [12]. To compensate for the cookstove trial's clustered design 1481 participants were recruited.

For the current follow-up, sample size estimates were informed by reported rates of FEV_1_ decline in US, UK and Australian cohort studies [[22], [23], [24]]. We estimated the minimum sample required to detect an annual FEV_1_ change ≥ 17 ml to be 146 (73 men, 73 women) per year with 90% power, and α = 0.05, and included 10% adjustment for attrition and 20% for important covariates in the analysis. Given the five years between 2014 and 2019, the expected difference would be fivefold, we therefore anticipated a minimum of 730 participants (365 men, 365 women) would suffice.

Global Lung Initiative 2012 reference values for African-American ethnicity were used [25]. Absolute FEV_1_ and FVC were included as continuous variables in longitudinal analyses. Each participant's spirometry was categorised into patterns consistent with clinical diagnoses: ‘normal,’ pre-bronchodilator FEV_1_ ≥ lower limit of normal (LLN), FVC ≥ LLN, FEV_1_/FVC ≥ LLN; ‘COPD,’ post-bronchodilator FEV_1_/FVC < LLN with no/insignificant bronchodilator reversibility; ‘asthma,’ pre-bronchodilator FEV_1_/FVC < LLN and significant reversibility (post-bronchodilator improvement in FEV_1_ ≥ 12% and ≥ 200 ml); and ‘restriction,’ post-bronchodilator FVC < LLN, FEV_1_/FVC ≥ LLN [9,10].

Primary outcome measures were lung function (FEV_1_, FVC) and HRQoL score. Rate of change of FEV_1_, and FVC were expressed as ml/year and *z*-score/year. Rate of change in HRQoL score associated with changes in the lung function were also estimated. Clinical considerations, analysis of variance and simple linear regression methods identified the following explanatory variables for inclusion in multivariable analysis: follow-up time, age, height, BMI, sex, previous TB diagnosis, smoking, years of schooling, clinical spirometry patterns, reported respiratory diagnoses (any of asthma, emphysema, chronic bronchitis, COPD).

Linear mixed-effects models were used to analyse lung function using all the 2014 and 2019 data. To investigate associations with rates of FEV_1_ and FVC, multiplicative terms were individually included in models: time*diagnosed asthma, time*diagnosed COPD, time*previous TB, time*smoking history, time*spirometry consistent with asthma, time*spirometry consistent with COPD, time*restrictive spirometry. Sensitivity analyses included those participants with lung function from both 2014 and 2019. HRQoL data were only collected in 2014 and 2019. Linear mixed-effects models with robust standard errors were used to analyse HRQoL scores because of they have an increasingly skewed distribution. Beta regression models with their important explanatory variables were used to conduct sensitivity analysis of HRQoL to assess validity of the mixed-effects model with robust standard errors. In all mixed-effects models, random effects were accounted for at the individual level whilst the explanatory fixed-effects were selected sequentially with the optimum model fit by likelihood ratio and deviance testing under maximum likelihood estimation. Statistical analysis was conducted using R v3.6.0. All significance tests were two-tailed, with *p* < 0.05 considered significant.

### 2.5 Role of the funding source

The funder of the study had no role in the study design, data collection, data analysis, data interpretation, or writing of the report. MWN, PB, GD, AO, LN, and JR had full access to all the data in the study. All the authors had final responsibility for the decision to submit for publication.

## 3 Results

**Fig. 1:**
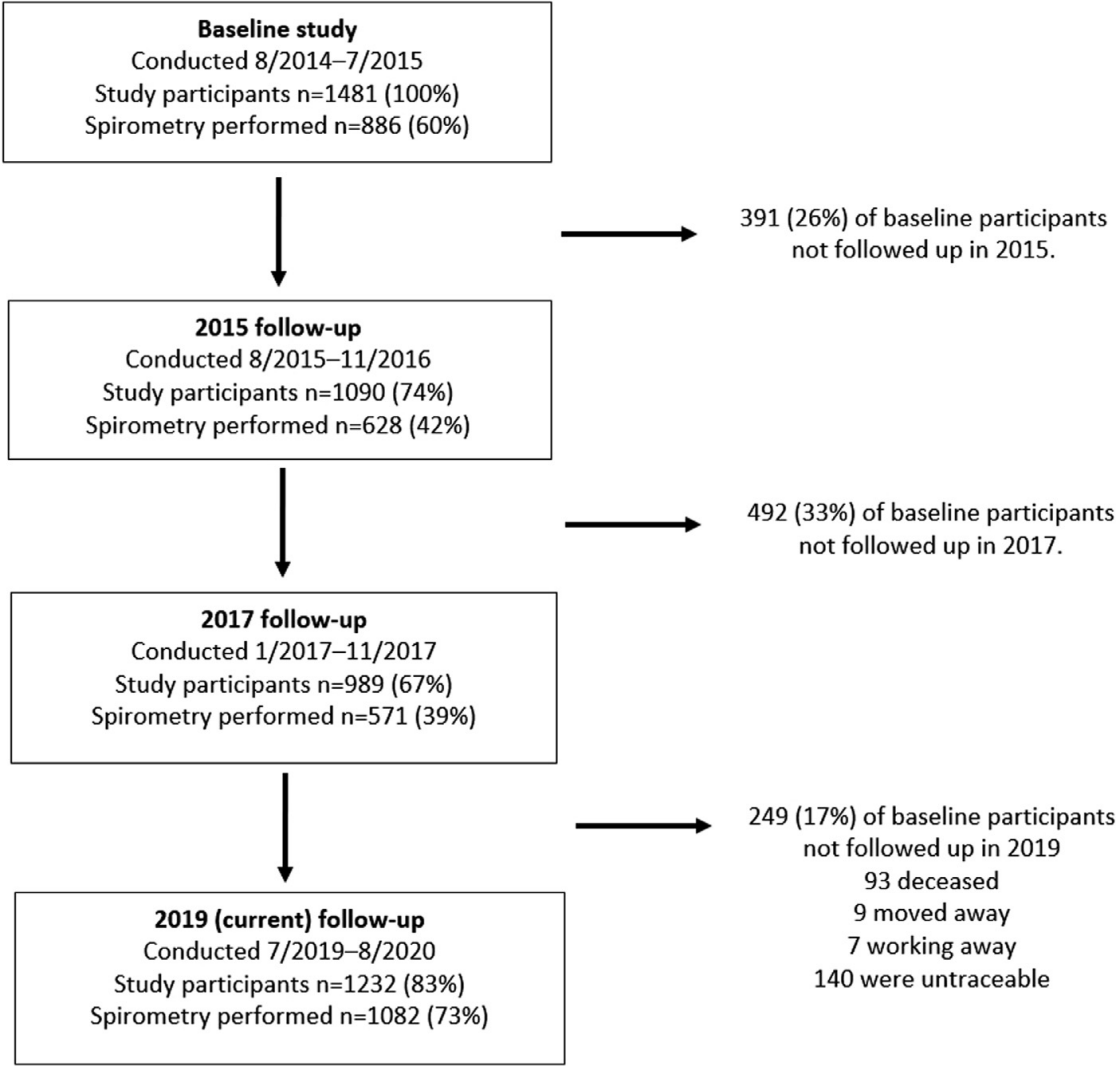
Participants in the various follow-up phases of the Chikwawa lung health cohort.

Between July 2019 and August 2020, 1232 (83%) of the 1481 original participants were assessed. We were unable to follow-up 249: 93 (6.3%) had died, 9 (0.6%) had moved away, 7 (0.5%) were working away, and no information was available for 140 (9.5%). Fig. 1 outlines participant numbers in each follow-up.

Mean (SD) participant age in 2019 was 49.5 (17.0) years, 278 (23%) had ever smoked, and 724 (59%) were women (Table 1). A greater proportion of the 2014 cohort participated in 2019 (*n* = 1232 (83%)) than in 2015 (*n* = 1090 (74%)) or 2017 (*n* = 989 (67%)). Comparison of the 2014 characteristics of those participating or not participating in 2019 (supplement Table E1) indicated that those participating in 2019 were older (mean difference [MD] (95% CI): 7.9 years (5.8, 9.9)), less educated (MD: 1.2 years, 0.7, 1.7), less likely to have acceptable spirometry (percentage difference (95% CI): 12.1% (5.5, 18.7%)), had reduced FEV_1_ (MD (95% CI): 3.71%, (0.02, 7.40%)), and reduced HRQoL score (MD (95% CI): 0.03, (0.01, 0.05)). However, participation in 2019 was not significantly associated with sex, smoking history, respiratory symptoms, or diagnoses.

**Table 1:** Characteristics of study participants at baseline (2014) and in 2019.

	Baseline 2014 (*n* = 1481)	2019 follow-up (*n* = 1232)
Women, *n* (%)	844 (57.0%)	724 (58.8%)
		
Age (years, mean (SD))	43.9 (17.8)	49.5 (17.0)
Grouped age (years), *n* (%)		
< 39	685 (46.3%)	403 (32.7%)
40–49	258 (17.4%)	268 (21.8%)
50–59	217 (14.7%)	213 (17.3%)
60–69	161 (10.9%)	167 (13.6%)
> 70	160 (10.8%)	181 (14.7%)
		
BMI (kg/m^2^), mean (SD))	21.6 (3.46)	21.8 (3.93)
BMI, *n* (%)		
Underweight (< 18.5)	182 (13.9%)	219 (17.8%)
Normal weight (≥ 18.5; < 25.0)	950 (72.8%)	795 (64.4%)
Overweight (≥ 25.0; < 30.0)	133 (10.2%)	161 (13.1%)
Obese (≥ 30.0)	40 (3.1%)	58 (4.7%)
		
Never smoked	1154 (77.9%)	954 (77.4)
		
Years of schooling completed, mean (SD)	4.21(4.09)	3.80(3.94)
Highest level of education completed		
None	485 (32.7%)	477 (38.7%)
Primary School	758 (51.2%)	576 (46.8%)
High School	232 (15.7%)	165 (13.4%)
College & University	4 (0.27%)	14 (1.14%)
Unknown	2 (0.14%)	0 (0.00%)

The number of participants providing acceptable spirometry in 2019 (*n* = 1082) was greater than in 2014 (*n* = 886), 2015 (*n* = 628) and 2017 (*n* = 571), while 675 participants had acceptable spirometry in both 2014 and 2019 (Supplement Table E5). Participants providing acceptable spirometry in 2019 were significantly older, had more years of schooling and were less likely to report coughing, but did not differ significantly for sex, smoking, or symptoms of wheeze, breathlessness or sputum expectoration when compared with participants with no/unacceptable spirometry, (Supplement Table E2).

### 3.1 Symptoms

There was an increase in reported respiratory symptoms between 2014 and 2019 (Table 2); the proportion difference (95% CI) for ‘any respiratory symptom’ was 14.7% (11.6, 17.8%); ‘cough’ 12.5% (9.6, 15.5%); ‘sputum expectoration’ 7.8% (5.8, 9.7%); ‘wheezing’ 5.3% (3.7, 7.0%); ‘dyspnoea’ 4.0% (2.5, 5.6%). Participants also reported more diagnosed asthma, emphysema, or COPD 2.8% (1.0, 4.6%) and previous TB 3.8% (2.1, 5.6%). Restricting analysis to participants providing respiratory symptom data in both 2014 and 2019 made little difference to the magnitude of the observed differences (Supplement Table E3).

**Table 2:** Changes in symptom prevalence, diagnosed respiratory disease and lung function between 2014 and 2019.

	Baseline 2014 (*n* = 1481)	2019 follow-up (*n* = 1232)
*Symptoms*		
Any respiratory symptoms, *n* (%)	203 (13.7%)	350 (28.4%)
Cough most days of the month for ≥ 3 months of the year, *n* (%)	167 (11.3%)	294 (23.8%)
Usually brings up phlegm from chest, *n* (%)	39 (2.6%)	128 (10.4%)
Wheezing/whistling in chest in the past 12 months, *n* (%)	63 (4.3%	85 (6.9%)
Wheezing/whistling in chest in the past 12 months in the absence of a cold, *n* (%)	24 (1.6%)	60 (4.9%)
Shortness of breath when hurrying on the level or walking up a slight hill, *n* (%)	23 (1.6%)	69 (5.6%)
Breathing problems interfere with usual daily activities, *n* (%)	44 (3.0%)	44 (3.6%)
*Diagnosed lung disease*		
Diagnosed asthma, *n* (%)	51 (3.4%)	56 (4.5%)
Diagnosed COPD, *n* (%)	1 (0.1%)	11 (0.9%)
Diagnosed asthma, emphysema, chronic bronchitis, or COPD, *n* (%)	59 (4.0%)	84 (6.8%)
Previous TB, *n* (%)	47 (3.2%)	87 (7.0%)
		
*Spirometry*	*(n* *=* *886)*	*(n* *=* *1082)*
FEV_1_ (ml); mean (SD)	2645 (658)	2279 (723)
FVC (ml); mean (SD)	3283 (739)	2983 (835)
FEV_1_,%predicted; mean (SD)	97.8% (16.8%)	91.8% (19.3%)
FVC,%predicted; mean (SD)	101% (15.6%)	98.8% (19.8%)
FEV_1_/FVC; mean% (SD)	80.5% (8.5%)	76.1% (11.0%)
		
Obstruction		
FEV_1_/FVC<LLN *n* (%)*	84 (9.5%)	189 (17.5%)
FEV_1_/FVC<0.70 *n*(%)	82 (9.3%)	232 (21.4%)
Mild: FEV_1_≥80% predicted *n* (%)	47 (5.3%)	106 (9.8%)
Moderate: 50%≤FEV_1_<80% predicted *n* (%)	26 (2.9%)	103 (9.5%)
Severe: 30%≤FEV_1_<50% predicted *n* (%)	8 (0.9%)	17 (1.6%)
Very severe: FEV_1_<30% predicted	1 (0.1%)	6 (0.6%)
		
Restriction		
FVC <LLN *n* (%)	44 (5.0%)	91 (8.4%)
		
Spirometry clinical pattern		
Normal	702 (79.2%)	748 (68.2%)
COPD*	61 (6.9%)	134 (12.4%)
Asthma	23 (2.6%)	40 (3.7%)
Restriction	34 (3.8%)	52 (4.8%)
Unclassified not normal spirometry pattern	66 (7.4%)	108 (10.0%)

⁎ discrepancy reflect differences numbers for those with acceptable post-bronchodilator spirometry and those with acceptable pre and post bronchodilator spirometry.

### 3.2 Lung function changes

Comparison of spirometry data between 2014 and 2019 indicated that the unadjusted annual rate of FEV_1_ decline was 73.4 ml/year (95% CI): (61.0, 85.6; *p* < 0.0001) and for FVC 60.1 ml/year (46.0, 74.2; *p* < 0.0001) (supplement figure E1). When expressed as percent predicted and *z*-scores (supplement Table E4), the rate of FEV_1_ decline exceeded that of FVC and that predicted by GLI-2012. These rates of decline were reflected in the clinical patterns of lung function such that in 2019 a higher proportion had spirometry consistent with COPD (predominantly mild to moderate), however between 2014 and 2019 there was little difference in the proportion of participants with spirometry consistent with asthma and pure restriction (Table 2).

The lung function characteristics of participants with acceptable spirometry in both 2014 and 2019 (*n* = 675) and for those with data in 2014, 2015, 2017 and 2019 (*n* = 276) are presented in supplementary Tables E5, E6. In the 276 participants with acceptable spirometry in all four study phases, FEV_1_ declined by 55.2 ml/year (95% CI: 34.6, 75.8; *p* < 0.0001) and FVC by 47.2 ml/year (22.6, 72.0; *p* < 0.0001).

In the mixed-effects regression model (Table 3) of all participants with acceptable spirometry in 2014 and/or 2019 (model 1), adjusted FEV_1_ declined by 53.4 ml/year (95% CI): (49.0, 57.8; *p* < 0.0001) and FVC by 45.2 ml/year (39.2, 50.5; *p* < 0.0001). Reduced FEV_1_ and FVC were significantly associated with age (FEV_1_ *p* < 0.001; FVC *p* < 0.001), female sex (FEV_1_ *p* < 0.001; FVC *p* < 0.001), reducing height (FEV_1_ *p* < 0.001; FVC *p* < 0.001), reducing BMI (FEV_1_ *p* < 0.001, FVC *p* = 0.004), but not smoking history (FEV_1_ *p* = 1; FVC *p* = 0.803) or years of schooling (FEV_1_ *p* = 1; FVC *p* = 0.251). Reported previous TB was associated with reduced FEV_1_(*p* < 0.001) and FVC(*p* < 0.001), whereas reported diagnosed asthma was associated with reduced FEV_1_(*p* = 0.014) but not FVC(*p* = 0.453), a reported diagnosis of emphysema (FEV_1_ *p* = 1; FVC *p* = 1), chronic bronchitis (FEV_1_ *p* = 1; FVC *p* = 1), or COPD (FEV_1_ *p* = 0.830; FVC *p* = 0.485) was not associated with FEV_1_ or FVC. When compared to those with normal lung function, spirometry patterns consistent with COPD or restriction were associated with reduced FEV_1_(*p* < 0.001) and FVC (*p* < 0.001) and spirometry consistent with asthma was associated with reduced FEV_1_ (*p* < 0.001) but not FVC (*p* = 0.260). None of the included interaction terms were significantly associated with FEV_1_ and FVC indicating that rates of FEV_1_ and FVC decline were not associated with diagnosed asthma (FEV_1_ *p* = 0.429; FVC *p* = 0.619), previous TB (FEV_1_ *p* = 0.091; FVC *p* = 0.092), smoking history (FEV_1_ *p* = 0.910; FVC *p* = 0.108), or spirometry consistent with asthma (FEV_1_ *p* = 0.731; FVC *p* = 0.160), COPD (FEV_1_ *p* = 0.900; FVC *p* = 0.977), or restriction (FEV_1_ *p* = 0.502; FVC *p* = 0.531). Inclusion of height squared and cookstove intervention allocation status between 2014 and 2015 in the models revealed no associations between the cookstove intervention or height squared on FEV_1_, FVC or their rates of decline. A sensitivity analysis of participants with acceptable spirometry in both 2014 and 2019 (model 2) indicated that adjusted FEV_1_ declined by 46.1 ml/year (95% CI): (22.7, 69.0; *p* < 0.0001) and FVC by 42.4 ml/year (37.7, 47.0; *p* < 0.0001). The parameter estimates for FEV_1_ and FVC *z*-scores are presented in supplement Table E7.

**Table 3:** Linear Mixed Effects Modelling applied to FEV1 and FVC data from participants in 2014 and 2019, statistically significant associations.

	FEV_1_ (ml)		FVC (ml)	
	Model 1$$	Model 2££	Model 1$$	Model 2££
	Estimate (95% CI)	Estimate (95% CI)	Estimate (95% CI)	Estimate (95% CI)
Time to follow-up (per year)	−53.5 (−57.8, −49.0)	−46.1 (−69.0, −22.7)	−45.2 (−50.5, −39.2)	−42.4 (−47.0, −37.7)
Age (per year)	−16.2 (−17.3, −15.0)	−15.6 (−17.0, −13.9)	−10.2 (−11.7, −8.6)	−9.3 (−11.1, −7.3)
Height (per cm)	26.4 (24.0, 29.2)	25.2 (22.2, 28.1)	34.1 (30.6, 37.6)	32.2 (28.3, 36.0)
Sex (female)	−465 (−513, −420)	−488 (−542, −433)	−630 (−686, −569)	−642 (−714, −573)
BMI (per kg/m^2^)	12.3 (7.2, 17.3)	12.6 (6.0, 18.1)	9.5 (2.4, 15.8)	8.8 (1.0, 16.5)
				
Previous TB	−213 (−295, −140)	−198 (−292, −105)	−174 (−276, −84.5)	−189 (−313, −76.1)
Diagnosed asthma	−113 (−196, −32.7)	−123 (−214, −32.7)		
				
Clinical spirometry pattern (compared to normal)				
COPD	−386 (−428, −346)	−324 (−374, −279)	−146 (−196, −94.4)	−88.9 (−146, −27.9)
Asthma	−627 (−754, −491)	−497 (−669, −312)		
Restriction	−691 (772, −612)	−673 (−773, −753)	−813 (−911, −705)	−754 (−877, −627)

TB, Tuberculosis; BMI, body mass index; FVC, forced vital capacity; FEV_1_, forced expiratory volume in 1 s.

FEV_1_ model: 88% of the variability of FEV_1_ accounted for in the final mixed-effects regression model of which fixed-effects accounts for 70% of the variation.

FVC model: 86% of the variability of FVC accounted for in the final mixed-effects regression model of which fixed-effects accounts for 62% of the variation.

$$ Models 1 include FEV_1_ and FVC data from all the participants with acceptable spirometry in 2014 (*n* = 886) and/or 2019 (*n* = 1082).

££ Models 2, sensitivity analysis, include FEV_1_ and FVC data from participants with acceptable spirometry in 2014 and 2019 (*n* = 675).

### 3.3 Changes in health-related quality-of-life

Between 2014 and 2019 there was a significant decline in unadjusted mean (SD) HRQoL utility score from 0.873 (0.133) to 0.790 (0.116) (*p* < 0.0001) and the proportion reporting perfect health (score of 1) declined from 39% (*n* = 582) to 2% (*n* = 23) (Supplement Fig. E2). Reduced HRQoL in 2014 and 2019 was associated with reported respiratory symptoms and previously diagnosed TB (Supplement Table E8). Diagnosed emphysema, chronic bronchitis or COPD was associated with reduced HRQoL in 2019 but not 2014, diagnosed asthma was not associated with HRQoL (Table 4). Spirometry consistent with asthma was associated with reduced HRQoL (MD, (95% CI): 0.080 (0.021, 0.131; *p* = 0.0029), as was spirometry consistent with COPD (0.037, [0.020, 0.055, *p* < 0.0001]) and pure restriction (0.055, [0.020, 0.089, *p* = 0.0003]) (Supplement Fig. E3).

**Table 4:** HRQoL scores of participants in 2014 and 2019 associations with respiratory symptoms and diagnosed respiratory diseases.

	2014			2019		
	HRQOL scoremean, (SD)			HRQOL scoremean, (SD)		
	Yes	No	p	Yes	No	p
*Symptoms*						
Any respiratory symptoms	0.805 (0.138) (*n* = 203)	0.894 (0.122) (*n* = 1163)	< 0.001	0.752 (0.133) (*n* = 350)	0.804 (0.100) (*n* = 882)	< 0.001
Cough ≥ 3 months of the year	0.811 (0.141) (*n* = 167)	0.880 (0.130) (*n* = 1314)	< 0.001	0.758 (0.130) (*n* = 294)	0.800 (0.109) (*n* = 938)	< 0.001
Usually brings up phlegm from chest	0.788 (0.153) (*n* = 39)	0.874 (0.132) (*n* = 1442)	0.009	0.731 (0.145) (*n* = 128)	0.796 (0.110) (*n* = 1104)	< 0.001
Wheezing/whistling in chest in the past 12 months, *n* (%)	0.790 (0.147) (*n* = 63)	0.876 (0.131) (*n* = 1418)	< 0.001	0.683 (0.142) (*n* = 85)	0.797 (0.104) (*n* = 1147)	< 0.001
Wheezing in the past 12 months in the absence of a cold	0.789 (0.130) (*n* = 24)	0.874 (0.133) (*n* = 1457)	0.004	0.676 (0.140) (*n* = 60)	0.795 (0.111) (*n* = 1172)	< 0.001
Shortness of breath when hurrying on the level or walking up a slight hill.	0.759 (0.130) (*n* = 23)	0.888 (0.123) (*n* = 1310)	< 0.001	0.687 (0.124) (*n* = 69)	0.796 (0.112) (*n* = 1163)	< 0.001
Breathing problems interfere with usual daily activities.	0.792 (0.151) (*n* = 44)	0.875 (0.132) (*n* = 1437)	0.001	0.669 (0.160) (*n* = 44)	0.794 (0.112) (*n* = 1188)	< 0.001
						
*Diagnosed lung disease*						
Diagnosed asthma	0.824 (0.140) (*n* = 51)	0.874 (0.132) (*n* = 1430)	0.014	0.754 (0.149) (*n* = 56)	0.791 (0.114) (*n* = 1176)	0.069
Diagnosed, emphysema, chronic bronchitis, or COPD.	0.827 (0.142) (*n* = 59)	0.874 (0.132) (*n* = 1422)	0.013	0.727 (0.154) (*n* = 84)	0.794 (0.111) (*n* = 1148)	< 0.001
Previous TB	0.806 (0.142) (*n* = 47)	0.875 (0.132) (*n* = 1434)	0.002	0.733 (0.152) (*n* = 87)	0.794 (0.111) (*n* = 1145)	< 0.001

In the mixed-effects regression model (Table 5) reduced HRQoL was associated with time (*p* < 0.001), older age at baseline (*p* < 0.001) and female sex (*p* < 0.001) but not years of schooling (*p* = 0.807) or smoking (*p* = 0.085).

**Table 5:** Robustly fit linear mixed effects modelling applied to HRQoL data from participants in 2014 and 2019.

	HRQoL$$			
	Model A (Symptoms + spirometry clinical pattern)		Model B (Symptoms + spirometry)	
	Estimate(95% CI)	*P* valueΨ	Estimate(95% CI)	*p* valueΨ
Time to follow-up (2019 vs 2014)^a^	−0.083 (−0.093, −0.073)	< 0.001	−0.081 (−0.091, −0.071)	< 0.001
*Age group*^b^				
40–49 years	−0.002 (−0.014, 0.011)	0.819	−0.003 (−0.015, 0.010)	0.670
50–59 years	−0.023 (−0.037, −0.009)	0.001	−0.026 (−0.040, −0.011)	< 0.001
60–69 years	−0.020 (−0.036, −0.004)	0.013	−0.022 (−0.038, −0.006)	0.006
≥ 70 years	−0.074 (−0.092, −0.056)	< 0.001	−0.079 (−0.098, −0.060)	< 0.001
Sex (female)	−0.032 (−0.041, −0.022)	< 0.001	−0.031 (−0.041, −0.021)	< 0.001
*Symptoms*				
Cough ≥ 3 months of the year	−0.023 (−0.037, −0.009)	0.001	−0.023 (−0.037, −0.010)	0.001
Usually brings up phlegm from chest	−0.021 (−0.042, 0.001)	0.058	−0.021 (−0.042, 0.000)	0.047
Wheezing in the past 12 months in the absence of a cold	−0.062 (−0.087, −0.036)	< 0.001	−0.062 (−0.088, −0.036)	< 0.001
Shortness of breath when hurrying on the level or walking up a slight hill	−0.093 (−0.119, −0.066)	< 0.001	−0.091 (−0.117, −0.064)	< 0.001
*Diagnoses*				
Previous TB	−0.032 (−0.055, −0.009)	0.006	−0.026 (−0.049, −0.003)	0.028
*Spirometry pattern*^c^^,^&				
COPD	−0.009 (−0.021, 0.003)	0.152		
Asthma	−0.040 (−0.080, −0.001)	0.047		
Restrictive	−0.037 (−0.059, −0.013)	0.002		
*Spirometry*				
FEV_1_% predicted (per%)			0.0005 (0.000, 0.001)	0.034
FVC% predicted (per%)			0.0004 (−0.0003, 0.001)	0.583

Ψ Significance was determined using Satterthwaite approximations of degrees of freedom.Reference Category.

a Baseline study.

b < 40 years.

c Normal spirometry pattern.

& The spirometry pattern variable was added into the model instead of FEV_1_ variable.

$$ Models include HRQoL data from all the participants with acceptable spirometry (2014 *n* = 886, 2019 *n* = 1082).

The symptoms of dyspnoea, wheeze, and cough were independently associated with reduced HRQoL, with dyspnoea appearing to be of greatest magnitude and cough the least. Reduced HRQoL was associated with previous TB, but not diagnosed asthma, emphysema, chronic bronchitis, or COPD. HRQoL was beneficially associated with FEV_1_%predicted but not FVC. There were no significant interactions between time and diagnosed conditions (diagnosed asthma *p* = 0.714; diagnosed COPD = 0.221; previous TB *p* = 0.876) or spirometric patterns (spirometry consistent with COPD *p* = 0.646; spirometry consistent with asthma *p* = 0.409; spirometry consistent with restriction *p* = 0.204).

## 4 Discussion

In this first longitudinal study of HRQoL in relation to ventilatory function and chronic respiratory disease in sub-Saharan Africa, we report adjusted annual rates of FEV_1_ and FVC decline of 53.4 ml/year and 45.2 ml/year, respectively. More robust estimates of FEV_1_ and FVC decline from those participating in both 2012 and 2019 were 46.1 ml/year and 42.4 ml/year, respectively. These rates of decline were reflected in an increase in spirometry consistent with COPD from 9.5% in 2014 to 17.5% in 2019. In the same period diagnosed COPD increased from 0.1 to 0.9%. There was no change in reported diagnosis of asthma or in spirometry consistent with asthma or pure restriction. The second main finding was that HRQoL was adversely and independently associated with the symptoms of dyspnoea, wheeze, and cough, previously diagnosed TB, and declining FEV_1_.

The annual rate of FEV_1_ decline of 46–53 ml/year in this cohort is greater than the ‘normal’ 24–29 ml/year decline in healthy non-smokers in high income countries (HICs), but similar to the 40–55 ml/year decline in smokers of ≥ 15 cigarettes/day in HICs, and the 53 ml/year reported in an accelerated FEV_1_ decline trajectory group accounting for 48% of COPD in US and European cohorts [22,26]. The cause(s) of the relatively rapid rate of FEV_1_ decline in Malawian adults is unclear. Although comparable to smoking 15 cigarettes/day in HICs, smoking is unlikely to underlie the observed decline in the general Malawian because only 14% of participants currently smoked, with most smoking a few cigarettes a day. Previous studies of this cohort have reported no association between lung function (or rate of decline) and smoking status, cookstove intervention and personal pollution exposure (PM_2.5_, CO) [15], similar to the finding from our analysis. The relatively rapid rate of FEV_1_ decline may be a result of frequent pulmonary infections consequent upon genetic factors and/or dietary factors. Reduced FEV_1_ (but not rate of decline) was associated with diagnosed asthma, previous TB, and spirometry consistent with COPD, asthma, and restriction, suggesting that the differences in lung function had occurred prior cohort set up and were not a consequence of differential rates of FEV_1_ decline. This finding is consistent with reports from HICs that reductions in FEV_1_ in later adult life observed in COPD and asthma are largely a consequence of the tracking of suboptimal lung function from childhood into adulthood [23,24,27].

The 2015 and 2017 follow-ups of this cohort reported an FEV_1_ decline of 30.9 ml/year and for FVC 38.3 ml/year, comparable with rates reported in healthy non-smokers in HICs [15,22,26]. The most likely explanation for the disparity between the 2015/7 and 2019 follow-up (30.9 vs 53.4 ml/year) is the greater number participants with acceptable spirometry in 2019 (*n* = 1082) compared with 2014 (*n* = 886), 2015 (*n* = 628) and 2017 (*n* = 571). It is likely that the 53.4 ml/year rate of decline reported here is an over-estimate resulting from participants providing spirometry in 2019, but not 2014, who were older, current smokers and symptomatic. The analysis of participants with acceptable spirometry in both 2014 and 2019 supports this notion, even so the rate of FEV_1_ decline of 46.1 ml/year is still cause for concern. We did not observe the high rates of restriction reported in urban Malawi (38.6%) and the 2014 baseline study of this cohort (34.8%) [12], probably reflecting use of NHANES III predictive equations for white Americans, whereas we used African-American GLI–2012 predictive equations as recommended by ATS/ERS [16].

Our findings that HRQoL is adversely and independently associated with age, female sex, respiratory symptoms (dyspnoea, wheeze, cough), previous TB and declining FEV_1_ is consistent with other studies. A cross-sectional study of 50 Nigerian COPD patients reported that respiratory HRQoL (St Georges Respiratory Questionnaire) is adversely associated with age, female sex and dyspnoea, however the Nigerian study reported no associations with wheeze, cough, or FEV_1_, probably reflecting the smaller sample size than our study [28]. The present study differs from a report from BOLD that chronic bronchitis symptoms are associated with reduced HRQoL (SF-12) and the impact is greater than for asthma or COPD [29]. In the current study, dyspnoea and wheeze symptoms were more strongly associated with HRQoL than cough and phlegm symptoms and HRQoL was adversely associated with spirometry consistent with asthma but not COPD. These differences with BOLD may be a consequence of BOLD being of cross-sectional design. Some cross-sectional studies in Africa have reported reduced HRQoL in people with asthma and after TB treatment [30], [31], [32]. In the study reported here, a previous diagnosis of TB and spirometry consistent with asthma and wheezing symptoms were adversely associated with HRQoL.

In the current study in Malawi, the differences in HRQoL independently associated with dyspnoea, wheeze, previous TB and spirometry consistent with asthma or pure restriction exceeded the minimally important difference (MID) reported for the SF-6D instrument in longitudinal studies ((MID) (0.033, 95% CI: 0.029–0.037)) and the MIDs reported for SF-6D in people with COPD (0.011, (SD 0.09)) [33].

The present study has strengths and limitations. Strengths include the use of objective validated measures of ventilatory function and HRQoL to follow-up a cohort of adults randomly identified in Malawi. Although this cohort is largely representative of adults in rural Malawi, a limitation is that nonparticipants in the original 2014 study were younger and more likely to be male, anecdotally, younger men in rural villages are more likely to seek work in towns/cities. The high rate of follow-up of the cohort in 2019 increases confidence in the generalizability of our findings to other similar settings. Spirometry was performed in accordance with ERS/ATS guidelines [16], of high-quality and enabled identification of COPD independent of participant report. Limitations include biases e.g., recall, social desirability, consequent upon reliance on self-reports of symptoms, diagnoses, and exposures by participants. Further limitations include the relatively short 5 year follow-up and unavoidable use of UK tariffs embedded within SF–6D to generate HRQoL scores because appropriate and specific tariffs from relevant sub-Saharan settings have not been published. The choice of tariff does matter, and country-specific tariffs should be used where available. The study was observational and consequently we can only report associations and cannot exclude the possibility of residual confounding by factors associated with participant selection and participation.

In conclusion in this cohort study of adults living in rural Malawi the prevalence of COPD has increased by 1–2% a year and is associated with an annual rate of FEV_1_ decline greater than that reported in HICs, combined with lung function deficits present before recruitment that are likely to reflect early life influences and/or sequelae of TB. Respiratory symptom burden and sub-optimal lung function are independently associated with reduced HRQoL of magnitude greater than the minimal important difference. These findings justify further research into the aetiology, natural history, and most importantly sustainable diagnosis and management of chronic respiratory diseases in sub-Saharan Africa.

## Funding

This was funded by a New Investigator Research Grant from the Medical Research Council (Ref: MR/L002515/1), a joint Global Health Trials Grant from the Medical Research Council, UK Department for International Development and Wellcome Trust (Ref: MR/K006533/1, a US National Institutes for Health R56 Grant (Ref: R56ES023566) and by the National Institute for Health Research (NIHR) (project reference 16/136/35) using UK aid from the UK Government to support global health research. The views expressed in this publication are those of the authors and not necessarily those of the NIHR or the UK Department of Health and Social Care.

## Contributors

MWN drafted the study protocol, the first version of the manuscript, conducted the formal analysis and coordinated the present study in Malawi. PM, CC, SR, and RN collected the data for this study. KM, SBG and JB contributed to the set-up of the baseline study. MWN, PB, JR and GD verified the underlying data in this study. AO, LWN, KM and PB acquired the resources for this study. LWN, JR, AO, and GD contributed to the study protocol, formal analysis of this study and contributed to the manuscript in several rounds of review. All authors critically revised the manuscript. All authors read and approved the final manuscript for submission.

## Data sharing statement

A minimal anonymised dataset and data collection tools are available online [3]. Further information about the data can be obtained from the corresponding author (martin.njoroge@lstmed.ac.uk). All the data from the Chikwawa lung health cohort presented in this article are stored by the research group on safe servers at the Malawi Liverpool Wellcome Trust programme (MLW), Malawi and the BOLD centre at Imperial College London, UK and handled confidentially.

## Declaration of Competing Interest

We declare no competing interests.

## Appendix Supplementary materials

Image, application 1

Image, application 2

Image, application 3
