# Supplementary material for: Changing lung function and associated health-related quality-of-life: A five-year cohort study of Malawian adults
Source: eClinicalMedicine. 2021 Oct 18;41:101166. doi: 10.1016/j.eclinm.2021.101166 (PMC8529201; doi:10.1016/j.eclinm.2021.101166)
Supplement: Supplementary file 1 [file mmc1.docx]

**Appendix**

**Changing lung function and associated health-related quality-of-life: a five-year cohort study of Malawian adults.**

The IMPALA Consortium members:

Imelda Bates^1^, Emmanuel Addo-Yobo^2^, Brian Allwood^3^, Hastings Banda^4^, Amsalu Binegdie^5^, Muhwa Jeremiah Chakaya^6,7^, Asma El Sony^8^, Adegoke Falade^9^, Jahangir Khan^1,10^, Maia Lesosky^11^, Bertrand Mbatchou^12^, Hellen Meme^13^, Beatrice Mutayoba^14^, Nyanda Elias Ntinginya^15^, S Bertel Squire^1^, Miriam Taegtmeyer^1^, Rachel Tolhurst^1^, William Worodria^16^, Heather Zar^17^, Eliya Zulu^18^ and Lindsay Zurba^19^.

1. Liverpool School of Tropical Medicine, Liverpool, UK
2. College of Health Sciences, Kwame Nkrumah University of Science and Technology, Kumasi, Ghana.
3. Stellenbosch University and Tygerberg Hospital, Cape Town, South Africa.
4. Research for Equity And Community Health (REACH) Trust, Lilongwe, Malawi.
5. College of Health Sciences, Addis Ababa University, Addis Ababa, Ethiopia.
6. The International Union Against Tuberculosis and Lung Disease, Paris, France
7. Kenyatta University, Nairobi, Kenya.
8. Public Health Epidemiological Laboratory [Epi Lab] for Research and Development, Khartoum, Sudan.
9. College of Medicine and University College Hospital, Ibadan, Nigeria.
10. Karolinska Institutet, Stockholm, Sweden.
11. University of Cape Town, Cape Town, South Africa.
12. Douala General Hospital, Douala, Cameroon
13. Centre for Respiratory Diseases Research, Kenya Medical Research Institute, Nairobi, Kenya
14. National TB and Leprosy Programme, Dodoma, United Republic of Tanzania
15. NIMR-Mbeya Medical Research Centre, Mbeya, Tanzania
16. Infectious Diseases Research Collaboration, Mulago Hospital, Makerere University, Kampala, Uganda.
17. Red Cross Children's Hospital and Medical Research Council Unit on Child and Adolescent Health, University of Cape Town, Cape Town, South Africa.
18. African Institute for Development Policy (AFIDEP), Lilongwe, Malawi.
19. Education for Health Africa, Vereeniging, South Africa.
