# Supplementary material for: Changing lung function and associated health-related quality-of-life: A five-year cohort study of Malawian adults
Source: eClinicalMedicine. 2021 Oct 18;41:101166. doi: 10.1016/j.eclinm.2021.101166 (PMC8529201; doi:10.1016/j.eclinm.2021.101166)
Supplement: Supplementary file 2 [file mmc2.docx]

**Table E1: The 2014 baseline characteristics of study participants who did and did not participate in the 2019 follow-up of the Chikwawa lung health cohort.**

**Table E2: 2019 study comparisons of participants with acceptable spirometry and those with no/unacceptable spirometry.**

**Table E3:** **Changes in symptom prevalence and diagnosed respiratory disease in those with data in both 2014 and 2019.**

**Figure E1: FEV_1_ and FVC box and whisker plots for participants grouped into clinical respiratory diagnoses by their spirometry. Each box-plot shows the variation in the lung function measures within each spirometry diagnosis. Scatter points are outlier values. Upper whisker shows Q3 (75^th^ percentile) plus 1.5*Interquartile range (IQR). Lower whisker shows Q1 (25^th^ percentile) minus 1.5*IQR.**

**Table E4: Changes in ventilatory function between 2014 and 2019 expressed as GLI-2012 z-scores.**

**Table E5:** **Ventilatory function of the participants with acceptable spirometry in 2014 and 2019.**

**Table E6: Ventilatory function of the participants with acceptable spirometry in 2014, 2015, 2017 and 2019.**

**Table E7: Linear Mixed Effects Modelling applied to FEV_1_ and FVC data expressed as z-scores from participants in 2014 and 2019, statistically significant associations.**

**Figure E2: Histograms with their overlaid kernel density plot (solid line) of the HRQoL scores of all the participants in 2014 and 2019 (range 0 – 1). The coloured dashed lines are the mean value in each follow-up study while the black dashed line shows an HRQoL score of 1 (perfect health)**

**Figure E3: Box and whisker plots of HRQoL in relation to spirometric patterns of respiratory disease in 2014 and 2019. HRQoL scores range 0 – 1. Scatter points are outlier values. Upper whisker shows Q3 (75^th^ percentile) plus 1.5*Interquartile range (IQR). Lower whisker shows Q1 (25^th^ percentile) minus 1.5*IQR.**

**Table E8: HRQoL scores of participants in 2014 and 2019 associations with respiratory symptoms and diagnosed respiratory diseases for those with acceptable spirometry.**
