# Supplementary material for: Changing lung function and associated health-related quality-of-life: A five-year cohort study of Malawian adults
Source: eClinicalMedicine. 2021 Oct 18;41:101166. doi: 10.1016/j.eclinm.2021.101166 (PMC8529201; doi:10.1016/j.eclinm.2021.101166)
Supplement: Supplementary file 3 [file mmc3.docx]

**Supplemental material**

**Changing lung function and associated health-related quality-of-life: a five-year cohort study of Malawian adults.**

Martin W Njoroge, MSc*^1,2^, Patrick Mjojo, Dip^2^, Catherine Chirwa, BA^2^, Sarah Rylance, PhD^1,2^, Rebecca Nightingale, PhD^1^, Prof Stephen B Gordon, MD^1,2^, Prof Kevin Mortimer, PhD^1,3^, Prof Peter Burney, MD^4^, Prof John Balmes, MD^5,6^, Prof Jamie Rylance, PhD^1,2^, Angela Obasi, PhD^1^, Prof Louis W Niessen, PhD^1,4^, Prof Graham Devereux, PhD^1^ on behalf of the IMPALA consortium.

1. Liverpool School of Tropical Medicine, Liverpool, UK
2. Malawi Liverpool Wellcome Trust Programme, Blantyre, Malawi
3. Liverpool University Hospital NHS Foundation Trust, Liverpool, UK
4. National heart and Lung Institute, Imperial College, London, UK
5. University of California, San Francisco, USA
6. University of California, Berkeley, USA
7. John Hopkins Bloomberg School of Public Health, Baltimore, USA

*Corresponding author, Department of Clinical Sciences and international Public Health, Liverpool School of Tropical Medicine, Pembroke Place, Liverpool L3 5QA, UK.

Email: [martin.njoroge@lstmed.ac.uk](mailto:martin.njoroge@lstmed.ac.uk)

**Table E1: The 2014 baseline characteristics of study participants who did and did not participate in the 2019 follow-up of the Chikwawa lung health cohort.**

| **2014 outcome** | **Participant in 2019 study**  **(n=1232)** | **Did not take part in the 2019 study**  **(n=249)** | **P value** |
| --- | --- | --- | --- |
| Women, n(%) | 724 (58.8%) | 154 (61.8%) | 0.41 |
| Age (years, mean (SD)) in 2014 | 43.4 (17.3) | 35.5 (15.3) | <0.0001 |
| BMI (kg/m^2^), mean (SD)) | 21.8 (3.93) | 21.6 (2.88) | 0.41 |
| Never smoked n (%) | 949 (77.4%) | 191 (76.7%) | 0.98 |
| Years of schooling completed, mean (SD) | 4.11 (3.99) | 5.34 (4.10) | <0.0001 |
|  |  |  |  |
| ***2014 symptoms*** |  |  |  |
| Any respiratory symptoms, n(%) | 166 (13.5%) | 26 (10.4%) | 0.23 |
| Cough most days, ≥3 months of the year, n(%) | 138 (11.2%) | 23 (9.2%) | 0.44 |
| Usually brings up phlegm from chest, n(%) | 32 (2.6%) | 5 (2.0%) | 0.75 |
| Wheezing/whistling in chest in the past 12 months, n(%) | 50 (4.1%) | 10 (4.0%) | 1.00 |
| Wheezing/whistling in chest in the past 12 months in the absence of a cold, n(%) | 19 (1.5%) | 1 (0.4%) | 0.26 |
| Shortness of breath when hurrying on the level or walking up a slight hill, n(%) | 16 (1.3%) | 3 (1.2%) | 1.00 |
| Breathing problems interfere with usual daily activities, n(%) | 39 (3.2%) | 7 (2.8%) | 0.93 |
|  |  |  |  |
| ***2014 spirometry*** |  |  |  |
| Acceptable spirometry, n(%) | 712 (57.8%) | 174 (69.9%) | <0.0001 |
| FEV_1_, %predicted; mean (SD) | 98.8 (21.3) | 102.5 (22.4) | 0.05 |
| FVC, %predicted; mean (SD) | 102.1 (21.9) | 104.5 (21.7) | 0.19 |
| FEV_1_/FVC<LLN n (%) | 68 (5.5%) | 14 (5.6%) | 1.00 |
| FEV_1_/FVC< 70%; GOLD definition n(%) | 68 (5.5%) | 8 (3.2%) | 0.18 |
|  |  |  |  |
| ***2014 Quality of life score*** |  |  |  |
| SF-6D derived HRQoL* (mean, (SD)) | 0.877 (0.128) | 0.910 (0.116) | <0.0001 |

* Health related quality of life

**Table E2: 2019 study comparisons of participants with acceptable spirometry and those with no/unacceptable spirometry.**

| **2019 outcome** | **Acceptable spirometry**  **(n=1082)** | **No and unacceptable spirometry**  **(n=150)** | **P value** |
| --- | --- | --- | --- |
| Women, n(%) | 601 (55.5%) | 78 (52.0%) | 0.465 |
| Age (years, mean (SD)) | 49.3 (16.4) | 59.1 (12.6) | <0.001 |
| BMI (kg/m^2^), mean (SD)) | 21.7 (3.89) | 21.6 (3.84) | 0.673 |
| Never smoked | 835 (77.2%) | 106 (70.7%) | 0.098 |
| Years of schooling completed, mean (SD) | 3.85 (3.96) | 2.74 (3.43) | <0.001 |
|  |  |  |  |
| ***Symptoms*** |  |  |  |
| Any respiratory symptoms, n(%) | 304 (28.1%) | 38 (25.3%) | 0.541 |
| Cough on most days ≥3 months of the year, n(%) | 255 (23.6%) | 37 (67.3%) | <0.001 |
| Usually brings up phlegm from chest, n(%) | 109 (10.0%) | 10 (6.7%) | 0.239 |
| Wheezing/whistling in chest in the past 12 months, n(%) | 67 (6.2%) | 5 (3.3%) | 0.225 |
| Wheezing/whistling in chest in the past 12 months in the absence of a cold, n(%) | 47 (4.3%) | 4 (2.7%) | 0.455 |
| Shortness of breath when hurrying on the level or walking up a slight hill, n(%) | 56 (5.2%) | 5 (3.3%) | 0.439 |
| Breathing problems interfere with usual daily activities, n(%) | 33 (3.0%) | 3 (2.0%) | 0.648 |
|  |  |  |  |
| **Quality of life score** |  |  |  |
| SF-6D derived HRQoL (mean, (SD)) | 0.798 (0.110) | 0.798 (0.103) | 0.979 |

**Table E3:** **Changes in symptom prevalence and diagnosed respiratory disease in those with data in both 2014 and 2019.**

|  | **Baseline 2014**  **(n=1232)** | **2019 follow-up**  **(n=1232)** |
| --- | --- | --- |
| ***Symptoms*** |  |  |
| Any respiratory symptoms, n(%) | 166 (13.5%) | 350 (28·4%) |
| Cough on most days ≥3 months of the year, n(%) | 138 (11.2%) | 294 (23·8%) |
| Usually brings up phlegm from chest, n(%) | 32 (2.6%) | 128 (10·4%) |
| Wheezing/whistling in chest in the past 12 months, n(%) | 50 (4.1%) | 85 (6·9%) |
| Wheezing/whistling in chest in the past 12 months in the absence of a cold, n(%) | 19 (1.5%) | 60 (4·9%) |
| Shortness of breath when hurrying on the level or walking up a slight hill, n(%) | 16 (1.3%) | 69 (5·6%) |
| Breathing problems interfere with usual daily activities, n(%) | 39 (3.2%) | 44 (3·6%) |
|  |  |  |
| ***Diagnosed lung disease*** |  |  |
| Diagnosed asthma, n (%) | 42 (3.4%) | 56 (4·5%) |
| Diagnosed COPD, n (%) | 1 (0.1%) | 11 (0·9%) |
| Diagnosed asthma, emphysema, chronic bronchitis, or COPD, n (%) | 50 (4.1%) | 84 (6·8%) |
| Previous TB, n (%) | 37 (3.0%) | 87 (7·0%) |
|  |  |  |

**Figure E1: FEV_1_ and FVC box and whisker plots for participants grouped into clinical respiratory diagnoses by their spirometry. Each box-plot shows the variation in the lung function measures within each spirometry diagnosis. Scatter points are outlier values. Upper whisker shows Q3 (75^th^ percentile) plus 1.5*Interquartile range (IQR). Lower whisker shows Q1 (25^th^ percentile) minus 1.5*IQR.**


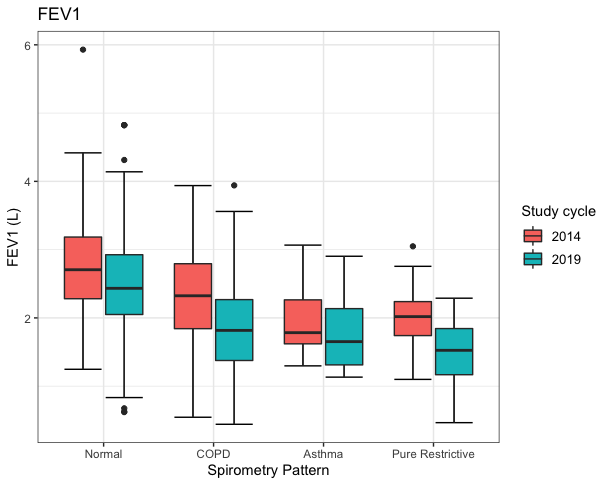


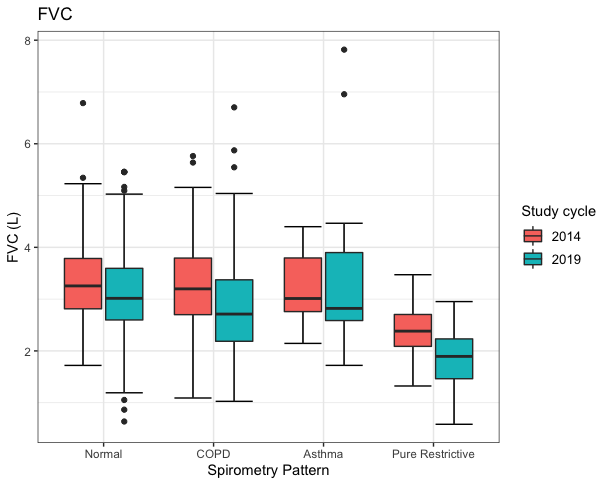


**Table E4: Changes in ventilatory function between 2014 and 2019 expressed as GLI-2012 z-scores.**

|  | **Baseline 2014**  **n=1481** | **2019 follow-up**  **n=1232** |
| --- | --- | --- |
| ***z-scores*** |  |  |
| FEV_1_ z-score; mean, (SD) | -0.140 (1.157) | -0.527 (1.275) |
| FVC z-score; mean, (SD) | 0.039 (1.091) | -0.135 (1.310) |
| FEV_1_/FVC z-score; mean, (SD) | -0.328 (0.996) | -0.663 (1.199) |
| FEV_1_/FVC<LLN n (%) | 84 (9.5%) | 189 (17.5%) |
| FVC<LLN n (%) | 44 (5.0%) | 91 (8.4%) |

Unadjusted annual decline in:

- FEV_1_ z-score was 0.077(95% CI): (0.056, 0.099; p<0.001)
- FVC z-score 0.035 (95% CI): (0.013, 0.056; p=0.002)

**Table E5:** **Ventilatory function of the participants with acceptable spirometry in 2014 and 2019.**

| **Spirometry** | **2014**  **(n=675)** | **2019**  **(n=675)** | **p value** |
| --- | --- | --- | --- |
| FEV_1_, %predicted; mean (SD) | 98.4 (16.4) | 93.6 (17.1) | <0.001 |
| FVC, %predicted; mean (SD) | 101.4 (15.5) | 99.1 (17.1) | 0.011 |
| FEV_1_/FVC; mean % (SD) | 80.6% (8.10) | 77.5% (9.24) | <0.001 |
|  |  |  |  |
| ***Obstruction*** |  |  |  |
| FEV_1_/FVC<LLN n (%) | 66 (9.8%) | 91 (13.5%) | 0.042 |
| FEV_1_/FVC<0.70 n(%) | 62 (9.2%) | 115 (17.0%) | <0.001 |
| Mild: FEV_1_≥80% predicted n (%) | 38 (5.6%) | 62 (9.2%) | <0.001 |
| Moderate: 50%≤FEV_1_<80% predicted n (%) | 20 (3.0%) | 46 (6.8%) |  |
| Severe: 30%≤FEV_1_<50% predicted n (%) | 3 (0.4%) | 6 (0.9%) |  |
| Very severe: FEV_1_<30% predicted | 1 (0.2%) | 1 (0.2) |  |
|  |  |  |  |
| ***Restriction*** |  |  |  |
| FVC <LLN n (%) | 28 (4.2%) | 47 (7.0%) | 0.032 |

**Table E6: Ventilatory function of the participants with acceptable spirometry in 2014, 2015, 2017 and 2019.**

| **Spirometry** | **2014**  **(n=276)** | **2015**  **(n=276)** | **2017**  **(n=276)** | **2019**  **(n=276)** | **p value^Ψ^** |
| --- | --- | --- | --- | --- | --- |
| FEV_1_, %predicted; mean (SD) | 97.4 (13.9) | 100 (16.1) | 97.5 (13.8) | 93.6 (17.1) | 0.003 |
| FVC, %predicted; mean (SD) | 99.5 (13.9) | 102 (15.9) | 99.4 (13.4) | 97.5 (14.9) | 0.106 |
| FEV_1_/FVC; mean % (SD) | 81.6% (6.55) | 82.0 (8.85) | 81.4 (7.14) | 78.7 (7.43) | <0.001 |
|  |  |  |  |  |  |
| ***Obstruction*** |  |  |  |  |  |
| FEV_1_/FVC<LLN n (%) | 16 (5.8%) | 21 (7.6%) | 22 (8.0%) | 31 (11.2%) | 0.033 |
| FEV_1_/FVC<0.70 n(%) | 14 (5.1%) | 18 (6.5%) | 19 (6.9%) | 34 (12.3%) | 0.004 |
| Mild: FEV_1_≥80% predicted n (%) | 9 (3.3%) | 13 (4.7%) | 13 (4.7%) | 19 (6.9%) |  |
| Moderate: 50%≤FEV_1_<80% predicted n (%) | 5 (1.8%) | 5 (1.8%) | 6 (2.2%) | 14 (5.1%) |  |
| Severe: 30%≤FEV_1_<50% predicted n (%) | 0 (0.0%) | 0 (0.0%) | 0 (0.0%) | 1 (0.4%) |  |
| Very severe: FEV_1_<30% predicted | 0 (0.0%) | 0 (0.0%) | 0 (0.0%) | 0 (0.0%) |  |
|  |  |  |  |  |  |
| ***Restriction*** |  |  |  |  |  |
| FVC<LLN n (%) | 13 (4.7%) | 13 (4.7%) | 10 (3.6%) | 19 (6.9%) | 0.363 |

**^Ψ^** 2019 vs 2014

Unadjusted annual decline in:

- FEV_1_ z-score was 0.057(95% CI): (0.022, 0.092; p=0.002)
- FVC z-score 0.036 (95% CI): (0.002, 0.071; p=0.039)

**Table E7: Linear Mixed Effects Modelling applied to FEV_1_ and FVC data expressed as z-scores from participants in 2014 and 2019, statistically significant associations.**

|  | **FEV_1_ (z-score)** | | **FVC (z-score)** | |
| --- | --- | --- | --- | --- |
|  | **Model 1^$$^** | **Model 2^££^** | **Model 1^$$^** | **Model 2^££^** |
|  | Estimate  (95% CI) | Estimate  (95% CI) | Estimate  (95% CI) | Estimate  (95% CI) |
| Time to follow-up  (per year) | -0.068  (-0.084,-0.053) | -0.061  (-0.077,-0.045) | -0.033  (-0.049, -0.017) | -0.033  (-0.049,-0.017) |
| Age (per year) | 0.012  (0.008,0.015) | 0.014  (0.010, 0.018) | 0.018  (0.014, 0.021) | 0.019  (0.015, 0.023) |
| Height (per cm) | -0.018  (-0.026,-0.011) | -0.024  (-0.032,-0.015) | -0.025  (-0.033,-0.018) | -0.031  (-0.039,-0.022) |
| Sex (female) | 0.379  (-0.505,-0.250) | -0.424  (-0.568, -0.274) | -0.468  (-0.595,-0.325) | -0.510  (-0.675,-0.359) |
| BMI (per kg/m^2^) | 0.032  (0.017,0.046) | 0.034  (0.0179,0.051) | 0.018  (0.003, 0.032) | 0.019  (0.001, 0.037) |
| Previous TB | -0.586  (-0.810,-0.386) | -0.596  (-0.864, -0.316) | -0.405  (-0.629, -0.182) | -0.447  (-0.723,-0.181) |
| Diagnosed asthma | -0.286  (-0.500,-0.075) | -0.333  (-0.569, -0.073) |  |  |
|  |  |  |  |  |
| Clinical spirometry pattern  (compared to normal) |  |  |  |  |
| COPD | -1.045  (-1.159,-0.928) | -0.881  (-1.016, -0.744) | -0.356  (-0.471, -0.246) | -0.234  (-0.375,-0.106) |
| Asthma | -1.568  (-1.926,-1.236) | -1.238  (-1.722,-0.707) |  |  |
| Restriction | -1.927  (-2.145,-1.725) | -1.822  (-2.076, -1.556) | -2.033  (-2.234, -1.753) | -1.816  (-2.111,-1.511) |

**^$$^** Models 1 include FEV_1_ and FVC data from all the participants with acceptable spirometry in 2014 (n=886) and/or 2019 (n=1082).

**^££^** Models 2, sensitivity analysis, include FEV_1_ and FVC data from participants with acceptable spirometry in 2014 and 2019 (n=675).

TB, Tuberculosis; BMI, body mass index; FVC, forced vital capacity; FEV_1_, forced expiratory volume in 1 second.

FEV_1_ z-score model: 66% of the variability of FEV_1_ accounted for in the final mixed-effects regression model of which fixed-effects accounts for 29% of the variation.

FVC z-score model: 65% of the variability of FVC accounted for in the final mixed-effects regression model of which fixed-effects accounts for 24% of the variation.

**Figure E2: Histograms with their overlaid kernel density plot (solid line) of the HRQoL scores of all the participants in 2014 and 2019 (range 0 – 1). The coloured dashed lines are the mean value in each follow-up study while the black dashed line shows an HRQoL score of 1 (perfect health)**


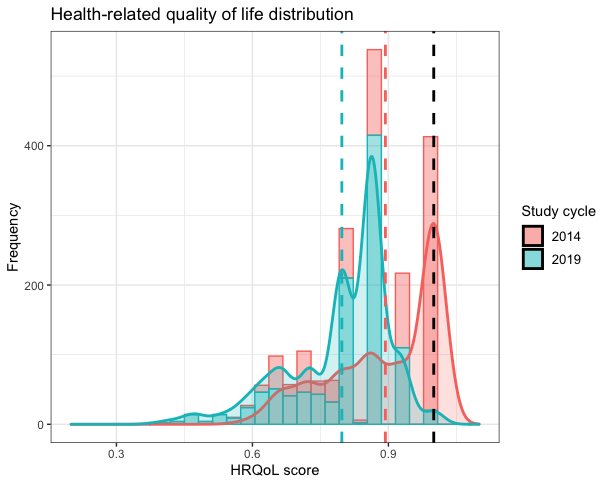


**Figure E3: Box and whisker plots of HRQoL in relation to spirometric patterns of respiratory disease in 2014 and 2019. HRQoL scores range 0 – 1. Scatter points are outlier values. Upper whisker shows Q3 (75^th^ percentile) plus 1.5*Interquartile range (IQR). Lower whisker shows Q1 (25^th^ percentile) minus 1.5*IQR.**


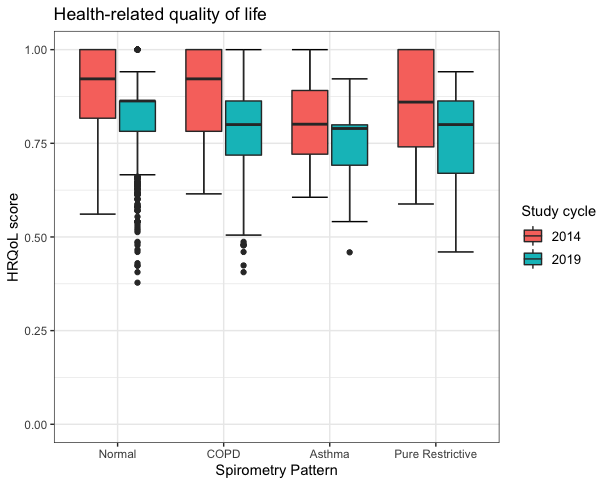


**Table E8: HRQoL scores of participants in 2014 and 2019 associations with respiratory symptoms and diagnosed respiratory diseases for those with acceptable spirometry.**

|  | **2014** | | | **2019** | | |
| --- | --- | --- | --- | --- | --- | --- |
|  | **HRQOL score**  **mean, SD** | |  | **HRQOL score**  **mean, SD** | |  |
|  | Yes | No | P value | Yes | No | P value |
| ***Symptoms*** | | | | | | |
| Any respiratory symptoms. | 0.831 (0.123)  (n=113) | 0.907 (0.111)  (n=719) | <0.001 | 0.764 (0.127)  (n=304) | 0.811 (0.100)  (n=778) | <0.001 |
| Cough ≥3 months of the year | 0.838 (0.125)  (n=87) | 0.899 (0.116)  (n=797) | <0.001 | 0.770 (0.123)  (n=255) | 0.807 (0.104)  (n=827) | <0.001 |
| Usually brings up phlegm from chest | 0.815 (0.132)  (n=23) | 0.895 (0.117)  (n=863) | 0.009 | 0.747 (0.143)  (n=109) | 0.804 (0.104)  (n=973) | <0.001 |
| Wheezing/whistling in chest in the past 12 months, n(%) | 0.809 (0.136)  (n=32) | 0.896 (0.116)  (n=852) | 0.001 | 0.697 (0.142)  (n=67) | 0.805 (0.104)  (n=1015) | <0.001 |
| Wheezing in the past 12 months in the absence of a cold | 0.811 (0.126)  (n=11) | 0.894 (0.118)  (n=873) | 0.054 | 0.688 (0.142)  (n=47) | 0.803 (0.106)  (n=1035) | <0.001 |
| Shortness of breath when hurrying on the level or walking up a slight hill. | 0.793 (0.113)  (n=11) | 0.901 (0.114)  (n=875) | 0.010 | 0.694 (0.121)  (n=56) | 0.803 (0.106)  (n=1026) | <0.001 |
| Breathing problems interfere with usual daily activities. | 0.818 (0.143)  (n=24) | 0.895 (0.117)  (n=862) | 0.015 | 0.684 (0.166)  (n=33) | 0.801 (0.106)  (n=1049) | <0.001 |
|  |  |  |  |  |  |  |
| ***Diagnosed lung disease*** | | | | | | |
| Diagnosed asthma. | 0.856 (0.132)  (n=29) | 0.895 (0.118)  (n=857) | 0.129 | 0.770 (0.139)  (n=48) | 0.799 (0.108)  (n=1034) | 0.155 |
| Diagnosed, emphysema, chronic bronchitis, or COPD. | 0.871 (0.130)  (n=33) | 0.894 (0.118)  (n=853) | 0.321 | 0.758 (0.142)  (n=68) | 0.801 (0.107)  (n=1014) | 0.017 |
| Previous TB | 0.834 (0.112)  (n=22) | 0.895 (0.118)  (n=864) | 0.020 | 0.749 (0.150)  (n=76) | 0.802 (0.106)  (n=1006) | 0.004 |
